# Virtual screening, molecular docking, MD simulation studies, DFT calculations, ADMET, and drug likeness of Diaza-adamantane as potential MAPK^ERK^ inhibitors

**DOI:** 10.3389/fphar.2024.1360226

**Published:** 2024-07-02

**Authors:** Davood Gheidari, Morteza Mehrdad, Foroozan Hoseini

**Affiliations:** Department of Chemistry, Faculty of Science, University of Guilan, Rasht, Iran

**Keywords:** virtual screening, molecular docking, MAPK^ERK^, Diaza-adamantane, MD simulation

## Abstract

**Introduction:** Multiple sclerosis (MS) is an autoimmune and inflammatory disease that destroys the protective coating of central nervous system (CNS) nerve fibers and affects over 2.8 million people worldwide. Despite several studies on new therapeutic targets and lead compounds, MS disease has limited treatment options. This condition may be caused by a complicated interaction of environmental and genetic variables. Studies showed that MS-associated microglial cells’ increased **MAPK^ERK^** activity may cause CNS inflammation and oligodendrocyte damage. Thus, screening for lead compounds that inhibit **MAPK^ERK^** may protect brain cells and slow disease progression.

**Methods:** The study aims to discover compounds that may inhibit **MAPK^ERK^** as a novel approach for protecting the nervous system in managing MS. The study includes in *silico* methods, such as virtual screening, molecular docking, Density-functional theory (DFT) investigations (using the B3LYP/6–31++G(d,p) basis set in a gas phase environment), drug likeness scores, and molecular dynamic (MD) simulations.

**Results and Discussion:**During the docking process with the **MAPK^ERK^** protein, it was shown that the ligand L_12_ receptor had the best binding affinity, with a docking score of -6.18 kcal/mol. To investigate the stability of the binding, a 100 ns MD simulation was performed on the complex formed by the **MAPK^ERK^** protein and L_12_. The receptor-ligand combination exhibited significant stability throughout the duration of the MD simulation. Additionally, the pharmacokinetic and drug-likeness properties of these ligands suggest that they have the potential to be considered viable candidates for future development in MS management.

## 1 Introduction

**FIGURE 1:**
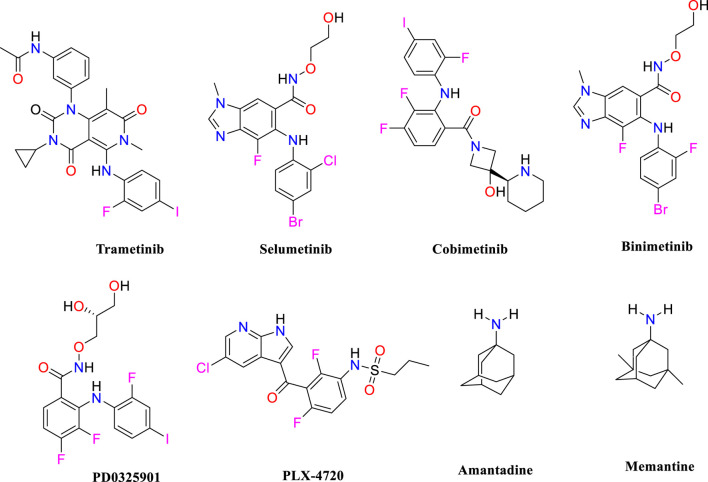
Chemical structure for the inhibition of MAPK^ERK^.

MS is an autoimmune condition that causes inflammatory demyelinating lesions in the CNS, affecting both white and gray matter (Kolahdouzan et al., 2019) and leading to progressive disability. The increasing frequency of MS is expected to rise, perhaps reaching a worldwide total of 2.8 million people (Soiza et al., 2018). The cause of MS is not completely understood; however, it is believed to be the consequence of a complicated interaction of environmental and genetic variables. Environmental variables include geographical location during pre-adult years, smoking behaviors, and the age of exposure to the Epstein-Barr virus, whereas genetic susceptibility entails the existence of 52 known alleles linked to the development of MS (Alrouji et al., 2023). An empirical finding indicates that increased MAPK^ERK^ activity in microglial cells linked to MS might be a factor in the development of localized inflammation in the CNS, in addition to regional oligodendrocyte dysfunction. Moreover, a clear causal connection has been established between the pathological characteristics of MS, such as inflammation, neurodegeneration, and demyelination, and the clinical manifestations of the disease. The excessive activity of MAPK^ERK^ in microglial cells, which can be observed in MS, indicates that these dysregulated microglia may have a harmful effect on oligodendrocytes, particularly in terms of myelination. The connection between microglia and oligodendrocytes highlights the importance of their interaction in the pathogenesis of MS (Peferoen et al., 2014). Prior studies have shown that impaired microglial activity negatively affects nearby oligodendrocytes (Pang et al., 2012). Oligodendrocytes are susceptible to substances generated by microglia due to MAPK^ERK^ activation (Li et al., 2008). Microglial MAPK^ERK^ activation leads to the induction of cytokines such as IL-1β and TNF-α (Kaur et al., 2013), which are associated with causing damage to adjacent oligodendrocytes and resulting in hypomyelination. Because oligodendrocytes play a vital role in supporting axons, any harm to oligodendrocytes is expected to affect axonal function (Fünfschilling et al., 2012). The negative impact of microglia on oligodendrocytes may explain the occurrence of local demyelination when microglia with an overexpression of MAPK^ERK^ are present in certain areas. Moreover, the activation of MAPK^ERK^ in microglia has been linked to the production of several pro-inflammatory mediators (Benveniste, 1997), which worsens sclerosis in the afflicted areas of the central nervous system. Although there have been many studies on prospective therapeutic targets and lead compounds for MS, the therapy choices for MS patients are still restricted. Hence, identifying lead molecules that may target the MAPK^ERK^ signaling pathway is crucial for therapeutic development. Pharmacological inhibitors that target this system have shown strong anti-inflammatory effects and provide potential for safeguarding neuronal cells from injury, which might help slow down the course of the illness. This study seeks to discover possible inhibitors of MAPK^ERK^ as a new neuroprotective approach for managing MS. As shown in Figure 1, trametinib (Pratt et al., 2020), selumetinib (Ciombor and Bekaii-Saab, 2015), cobimetinib (Han et al., 2016), binimetinib (Woodfield et al., 2016), PD0325901 (Suo et al., 2019), amantadine (Generali and Cada, 2014), and memantine (Turalde et al., 2021) have been identified as inhibitors of MAPK^ERK^.

The present study aimed to investigate the binding of these compounds to the MAPK^ERK^ receptor via molecular docking analysis, elucidating the interaction mechanism, and studying binding stability through MD simulation analysis. Additionally, ADMET characteristics were evaluated to identify pharmacological candidates with minimal toxicity.

## 2 Materials and methods

### 2.1 Database preparation

A subset of 137,972 natural products obtained from the ZINC12 database (Sterling and Irwin, 2012) underwent preparation procedures at a pH of 7.4, mirroring the physiological pH found in the human body. This procedure included the generation of every possible tautomer and ionized state (Release Schrödinger Suite, 2012). Following this, a meticulous screening protocol was implemented to assess each compound based on predefined criteria associated with drug-likeness properties, such as molecular weight (MW) not surpassing 500 Da, a LogP value of 5 or lower, a maximum of 10 hydrogen bond acceptors (HBA), no more than 5 hydrogen bond donors (HBD), a limitation of 10 rotatable bonds (RB), and a total polar surface area (TPSA) constrained to 140 Å^2^ or less. Following this screening, 15,000 compounds that met these criteria were screened.

### 2.2 Ligand preparation

The ligands used as input for the docking investigation were obtained from the virtual screening hits of the natural databases. Subsequently, ligands were integrated into the workstation, and energy was minimized by utilizing the OPLS3e force field in the LigPrep module of the program. This minimization aids in assigning bond order, adding hydrogens to ligands, and converting 2D structures to 3D structures for performing docking studies. The generated output file, including the best conformations of the ligands, was then used for docking studies (Schrödinger Release, 2017).

### 2.3 Protein preparation

The MAPK^ERK^ protein’s crystal structure (PDB ID: 4qte) underwent refinement *via* Schrödinger’s protein preparation wizard (Protein Preparation Wizard, 2017). This meticulous procedure ensured an elevated level of confidence in the accuracy of the protein structure. By converting it from its raw state to a refined form, the protein was ensured for subsequent molecular docking and dynamic studies, enhancing its suitability and reliability for comprehensive studies. The protein preparation process consisted of correcting bond orders, removing water molecules and other non-specific components from the crystal structure, and adding hydrogen atoms to the protein structure to modify the tautomeric and ionization states of amino acid residues. After adding the missing hydrogen, the system underwent restricted energy reduction using the OPLS 2005 force field to ensure high accuracy.

### 2.4 Molecular docking studies

The MAPK^ERK^ protein and its prepared ligand underwent docking using Schrödinger’s GLIDE module (Glide, 2017). This process aimed to improve binding affinity by aiding in the identification of specific structural motifs. The docking results were analyzed using XP descriptors, which provided useful details on a variety of intermolecular interactions. The Glide XP descriptor data facilitated the determination of energy associated with each pose, aiding in the optimization of the ligand conformation within the ligand-receptor complex. Before initiating the docking protocol, Schrödinger’s Glide Grid generation was employed to create a specific Glide Grid surrounding the co-crystallized ligand. Subsequently, this grid served as the framework for the docking procedure, facilitating the precise positioning and interaction analysis between the prepared ligand and the protein structures within the designated spatial coordinates.

### 2.5 In silico predicted physico-chemical parameters

The physico-chemical parameters were utilizing the Swiss ADME service (Daina et al., 2017) and ADMET lab 2.0 (ADMETlab 2.0, 2021). The anticipated parameters encompass several key molecular attributes: MW, HBD, HBA, the octanol/water partition coefficient (log P), aqueous solubility (Log S), predicted apparent Caco-2 cell permeability, human intestinal absorption propensity (HIA), plasma protein binding (PPB), and the tally of RB. These parameters collectively serve as crucial indicators for assessing the physicochemical properties, drug-likeness, and potential bioavailability of the compounds under investigation.

### 2.6 Docking validation

Validation of the docking study is necessary to ensure precise molecule docking. The precision of the molecular docking process is assessed using the Root Mean Square Deviation (RMSD). In order to calculate the RMSD, the co-crystallized ligand of 4qte was removed from it and then subjected to XP glide docking using the matching receptor grid. The obtained RMSD value, which was less than 2.0 Å, indicates the reliability of the docking study (Gheidari et al., 2023).

### 2.7 MD simulation

MD simulation was conducted utilizing Desmond through the Schrödinger-Maestro interface (Desmond, 2017). This enables the precise identification and prediction of ligand-receptor interactions and the validation of molecular docking results. MD simulations were performed by solving a substance in an explicit orthorhombic water box using the SPC water model. Sufficient Cl ions were added to the system in order to counterbalance the overall charge of the complex. The duration of the simulation was 100 ns. The NPT ensemble was utilized, maintaining a constant number of atoms, a pressure of 1.01325 bar, and a temperature of 300 K. The default thermostat was the 1.0‐ps interval Nose-Hoover chain method, and the default barostat was the 2.0‐ps interval Martyna-Tobias-Klein. The maestro simulation interaction diagram was used to assess the MD simulation.

### 2.8 Quantum chemistry via density-functional theory calculation

The DFT is used to ascertain the electron’s density and energy properties. The Gaussian 09W program performs calculations that illustrate the estimated structure of atoms, molecules, crystals, and surfaces, as well as their interactions (Frisch et al., 2009). The wavenumbers of the vibrations were determined by the use of the B3LYP method and a 6-31++G (d,p) basis set. The B3LYP functional is a valuable approach for accurately characterizing harmonic vibrational frequencies in molecules of small to medium size. The output verification files were analyzed using GuassView 6.0. Molecular orbital (MO) analysis is essential in quantum chemistry and has been used to thoroughly characterize chemical behavior. The highest occupied molecular orbital (HOMO) and lowest unoccupied molecular (LUMO) of a molecule are employed to characterize chemical properties, encompassing reactivity, stability, kinetics, hardness, softness, and electronegativity.

## 3 Results and discussion

### 3.1 Quantum chemistry through density-functional theory calculation

The optimization of ligands (**L**_**1**_ **-L**_**18**_) was initially conducted using the B3LYP/6-31++G (d,p) basis set in the gas phase, and the results obtained from this optimization are presented in Table 1.

**TABLE 1:** Geometric parameters of the ligands (**L**_**1**_ **-L**_**18**_).

S.No.	Ligand	Gas phase		
		Optimization energy (hartree)	Polarizability (α) (a.u.)	Dipole moment (Debye)
**1**	**L**_**1**_	−850.457	233.149	1.233
**2**	**L**_**2**_	−730.269	172.679	1.629
**3**	**L**_**3**_	−902.189	243.487	1.486
**4**	**L**_**4**_	−920.972	239.453	4.244
**5**	**L**_**5**_	−925.673	240.025	1.477
**6**	**L**_**6**_	−961.550	229.873	1.486
**7**	**L**_**7**_	−922.259	216.674	3.502
**8**	**L**_**8**_	−769.599	185.997	1.022
**9**	**L**_**9**_	−748.538	187.061	3.519
**10**	**L**_**10**_	−748.539	186.689	4.220
**11**	**L**_**11**_	−925.676	236.316	1.910
**12**	**L**_**12**_	−807.722	196.245	2.273
**13**	**L**_**13**_	−886.359	223.501	1.877
**14**	**L**_**14**_	−922.250	216.540	1.520
**15**	**L**_**15**_	−844.818	188.960	1.950
**16**	**L**_**16**_	−1128.472	190.657	2.579
**17**	**L**_**17**_	−733.912	174.660	1.719
**18**	**L**_**18**_	−863.071	201.204	4.500

The bold value L stands for ligand.

**FIGURE 2:**
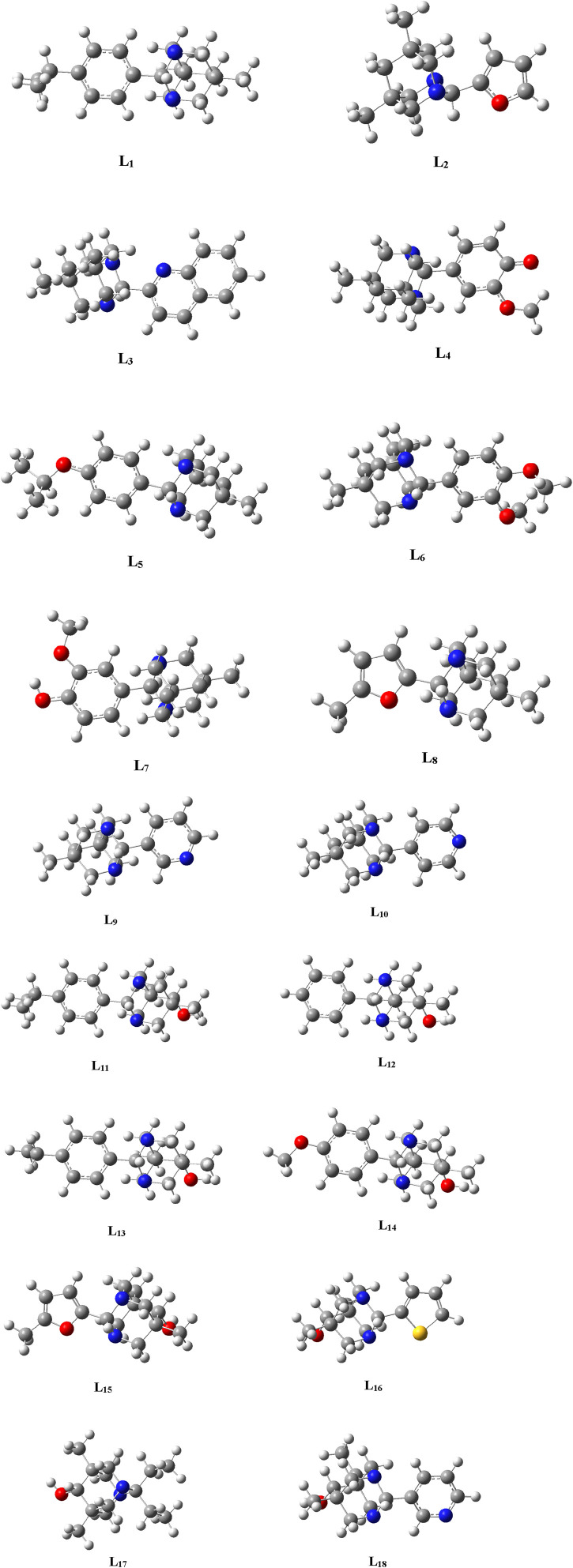
Optimized structures of the Ligands (**L**_**1**_ **-L**_**18**_) .

The geometries of the selected ligands underwent optimization until reaching the lowest energy gradient, confirming their status as true local minima, as no imaginary frequencies were detected. Figure 2 illustrates the optimized structures of the selected ligands.

**FIGURE 3:**
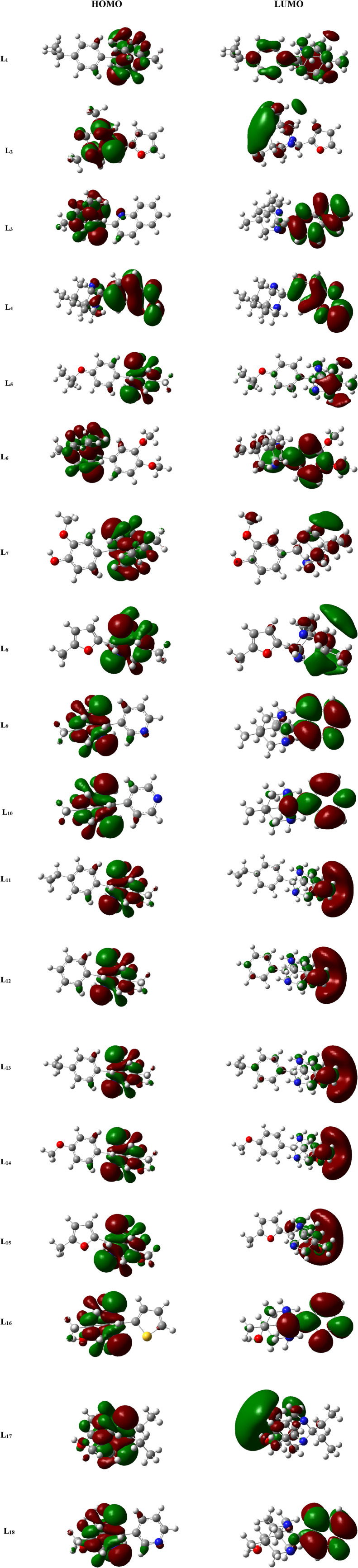
FMOs of the ligands (**L**_**1**_ **-L**_**18**_) .

The study of MO is very important in quantum chemistry, significantly enhancing our understanding and knowledge of chemical behavior. The HOMO and LUMO that are the chief molecular orbitals in a ligand are shown in Figure 3. The red and green color distributions represent the positive and negative phases, respectively, in the MO wave function.

**FIGURE 4:**
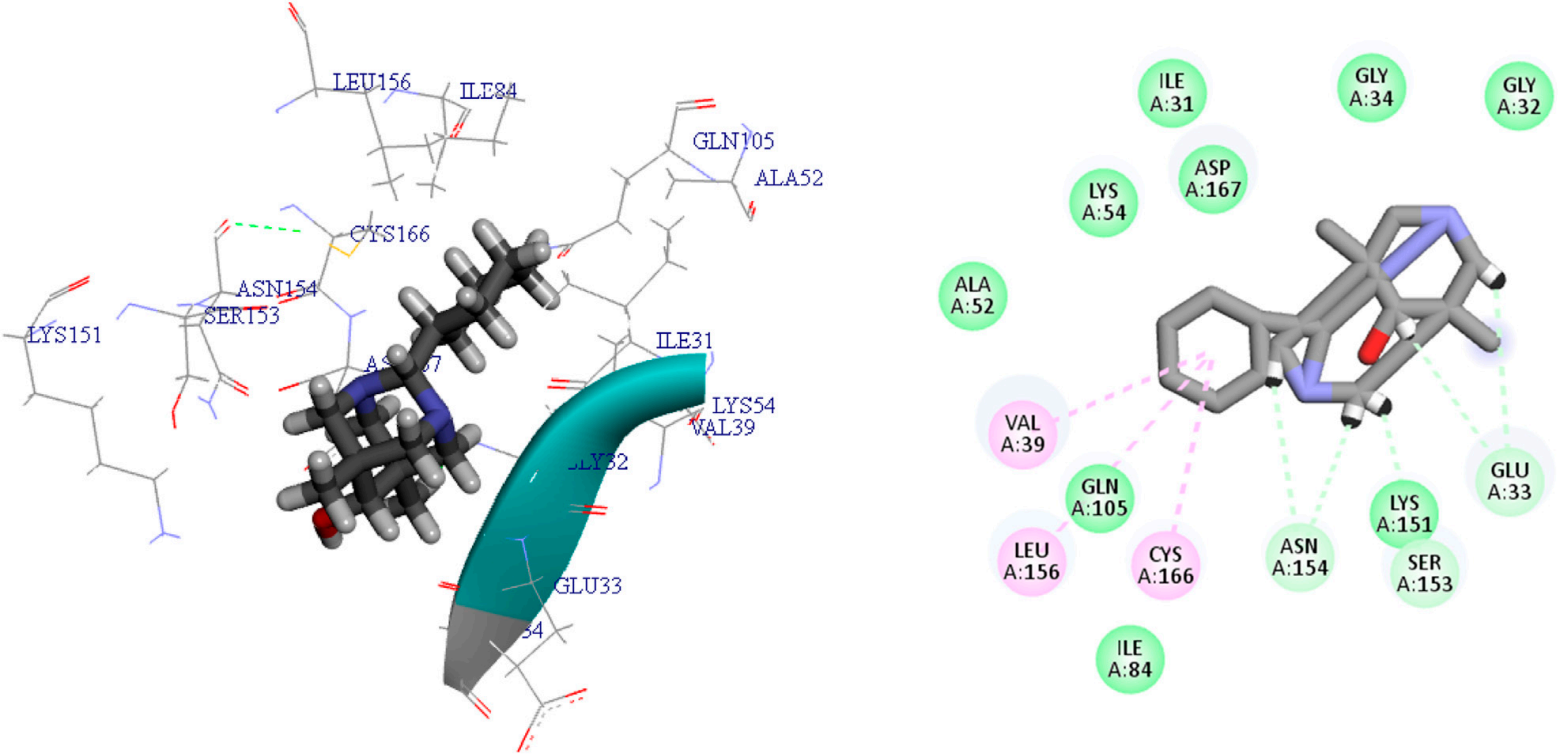
The 3D and 2D bindings mode of **L**_**12**_ into the active site of MAPK^ERK^.

The parameter hardness (*h*) quantifies the degree of hardness or softness exhibited by a molecule. Greater molecular softness correlates with enhanced reactivity. The atom’s electronegativity (*X*) indicates its ability to attract electron pairs towards its nucleus. The ligand **L**_**4**_, exhibiting the smallest HOMO–LUMO energy gap value of 2.4884 eV, suggests a heightened potential for chemical reactivity. Also, **L**_**4**_ demonstrates a low hardness value of 1.2442, positioning it as the softest ligand among all ligands. The greater electronegativity value of ligand **L**_**4**_ indicates that **L**_**4**_ has a strong ability to attract electrons and operates as a superior electrophile compared to other ligands. Ligands **L**_**3**_ and **L**_**10**_ exhibited favorable reactivity after **L**_**4**_, with energy gap values of 3.7621 eV and 4.7434 eV, respectively. Similarly, after **L**_**3**_, the ligands **L**_**11**_, **L**_**4**_, and **L**_**5**_ exhibit significant polarizability, with corresponding values of 236.316, 239.453, and 240.025. The energetic parameters of ligands (**L**_**1**_ **-L**_**18**_) are shown in Table 2.

**TABLE 2:** Energetic parameters of the ligands (**L**_**1**_ **-L**_**18**_).

Ligand	*E*_*HOMO*_ (eV)	*E*_*LUMO*_ (eV)	∆E _*gap*_ (eV)	Hardness(*η*)	Softness (*S*)	Electronegativity(*X*)	Electrophilicity(_*ψ*_)
**L**_**1**_	−5.4228	−0.3678	5.0549	2.5274	0.1978	2.8953	1.6584
**L**_**2**_	−5.4677	−0.3757	5.0919	2.5459	0.1963	2.9217	1.6765
**L**_**3**_	−5.3453	−1.5831	3.7621	1.8810	0.2658	3.4642	3.1898
**L**_**4**_	−5.5088	−3.0204	2.4884	1.2442	0.4018	4.2646	7.3086
**L**_**5**_	−5.3866	−0.3436	5.0430	2.5215	0.1982	2.8651	1.6278
**L**_**6**_	−5.4650	−0.3790	5.0860	2.5430	0.1966	2.9220	1.6787
**L**_**7**_	−5.4582	−0.4136	5.0446	2.5223	0.1982	2.9359	1.7086
**L**_**8**_	−5.4277	−0.3545	5.0732	2.5366	0.1971	2.8911	1.6476
**L**_**9**_	−5.6228	−0.8764	4.7464	2.3732	0.2106	3.2496	2.2249
**L**_**10**_	−5.6835	−0.9401	4.7434	2.3717	0.2108	3.3118	2.3123
**L**_**11**_	−5.4734	−0.4819	4.9915	2.4957	0.2003	2.9776	1.7763
**L**_**12**_	−5.5143	−0.5001	5.0141	2.5070	0.1994	3.0072	1.8035
**L**_**13**_	−5.4740	−0.4827	4.9913	2.4956	0.2003	2.9783	1.7772
**L**_**14**_	−5.4626	−0.4731	4.9894	2.4947	0.2004	2.9678	1.4588
**L**_**15**_	−5.4808	−0.4590	5.0217	2.5108	0.1991	2.9699	1.7564
**L**_**16**_	−5.6141	−0.5904	5.0237	2.5118	0.1990	3.1023	1.9157
**L**_**17**_	−5.3156	−0.4631	4.8525	2.4262	0.2060	2.8893	1.7204
**L**_**18**_	−5.6536	−0.8756	4.7779	2.3889	0.2092	3.2646	2.2306

### 3.2 Molecular docking of ligands with MAPK^ERK^ receptors

Schrödinger’s Ligand docking module utilizes grid-based ligand docking with energetics (GLIDE) (Glide, 2017) in three specific docking modes: high-throughput virtual screening (HTVS), standard precision (SP), and extra precision (XP) for docking and scoring. 15,000 compounds underwent screening by HTVS docking and scoring. A total of 50 ligands that had the highest G-Score with HTVS were further evaluated using SP docking and scoring. This was done to ensure that the docking postures were reliable and proper. To decrease false-positive findings, 18 ligands that were completely thriving were screened using XP docking and scoring. An analysis was conducted on the interactions of these protein-ligand complexes, and the outcomes are shown in Table 3. MAPK^ERK^’s active site’s amino acid residues, both bonding and non-bonding interactions with ligand **L**_**1**_ **–L**_**18**_, included Asn154, Ser153, Asp111, Tyr113, Gly32, Glu33, Gly34, Asp167, Leu156, Ile31, Cys166, Val39, Lys54, Tyr30, Lys114, Gln105, Ile84, Lys151, Asp111, Asp149, Ala35, Tyr36, Glu71, Ala52, and Lys117.

**TABLE 3:** Docking scores, and interaction of each ligands (**L**_**1**_ **-L**_**18**_).

Ligand	Docking scores (kcal/mol)	Interaction residue				
		Hydrogen bond		Van der walls	Hydrophobic	Unfavorable Donor-Donor
		H- bonding	C Bonding			
**L**_**1**_	−5.54		Asn154, Ser153	Asp111, Tyr113, Gly32, Glu33, Gly34, Asp167	Leu156, Ile31, Cys166, Val39, Lys54, Tyr30, Lys114	
**L**_**2**_	−5.64		Glu33, Ser153, Asn154, Gln105	Asp111, Gly34, Gly32, Ile84, Asp167, Lys151	Val39, Ile31, Leu156, Lys54, Cys166	
**L**_**3**_	−5.50	Asp111	Asp111, Glu33, Asn154, Ser153	Tyr30, Lys114, Ile31, Gly32, Tyr113, Leu156, Lys54, Asp167, Gly34	Val39, Cys166	
**L**_**4**_	−5.34		Gly34, Asn154, Ser153, Glu33, Asp167	Lys151, Asp149, Asp111, Gly32, Leu156, Lys54, Ala35, Tyr36	Cys166, Ile31, Val39	
**L**_**5**_	−5.06		Glu33, Asp111	Gly34, Gly32, Ile31, Tyr113, Ser153, Lys151, Asn154, Asp167, Glu71, Ile84, Gln105, Leu156	Val39, Cys166, Lys54, Lys114	
**L**_**6**_	−5.59	Lys54	Ser153, Asn154, Gln105, Glu33	Asp111, Lys151, Ile31, Leu156, Ala52, Glu71, Ile84, Asp167, Gly32, Gly34, Tyr113	Cys166, Val39	
**L**_**7**_	−5.34		Glu33, Asp167, Gly34, Ser153, Asn154	Asp111, Gly32, Tyr36, Ala35, Lys151, Asp149, Lys54, Leu156	Ile31, Cys166, Val39	
**L**_**8**_	−5.33		Asn154, Ser153, Asp167, Glu33	Lys54, Lys151, Asp149, Gly34, Gly32, Leu156, Asp111	Ile31, Val39, Cys166, Ala35	
**L**_**9**_	−5.39		Ser153, Asn154	Leu156, Lys54, Asp167, Asp149, Ala35, Gly34, Glu33, Gly32, Asp111	Val39, Cys166, Ile31	Lys151
**L**_**10**_	−5.37	Asp111	Ser153	Asp167, Lys54, Gly34, Glu33, Ile31, Gly32, Lys114, Tyr113, Asn154	Leu156, Cys166, Val39	
**L**_**11**_	−5.45		Glu33, Ser153, Asn154	Lys54, Asp167, Gly34, Tyr113, Lys114, Ile31, Gly32, Asp111	Tyr30, Cys166, Leu156, Val39	
**L**_**12**_	−6.18		Glu33, Ser153, Asn154	Gly34, Lys151, Gly32, Ile31, Ala52, Ile84, Gln105, Lys54, Asp167	Val39, Leu156, Cys166	
**L**_**13**_	−5.11		Ser153, Asn154, Glu33	Leu156, Gly32, Ile31, Asp111, Lys114, Lys117, Gly34, Asp167	Val39, Cys166, Tyr113	
**L**_**14**_	−5.15		Glu33, Ser153, Asn154	Lys54, Asp167, Gly34, Lys151, Tyr113, Gly32, Ile31	Cys166, Val39, Leu156	
**L**_**15**_	−5.90		Ser153, Asp167, Glu33, Asn154	Ala52, Ile31, Gly32, Asp111, Gly34, Lys151, Ile84, Gln105	Lys54, Leu156, Val39, Cys166	
**L**_**16**_	−5.64	Lys54	Glu33, Asn154, Ser153	Gly34, Ile31, Gly32, Lys151, Asp167, Ile84, Gln105	Val39, Leu156, Cys166, Lys54	
**L**_**17**_	−5.87		Asn154, Ser153, Glu33	Gly34, Asp167, Gln105, Asp111, Lys54, Gly32, Lys151,Ile31	Leu156, Ile31, Val39, Cys166	
**L**_**18**_	−5.11	Glu33	Asn154, Glu33, Ser153, Asp111	Lys54, Asp167, Lys151, Gly34, Tyr113, Lys114, Tyr30, Gly32, Ile31	Val39, Cys166, Leu156	

Ligands L12, L15, and L17 demonstrate enhanced efficacy relative to other ligands as a result of their favorable interactions inside the active site of the MAPKERK protein. The most favorable interactions occurred with L12, including five carbon H-bonds with the amino acid residues Asn154, Ser153, and Glu33. The computed bond lengths were 2.28 Å and 2.63 Å for Asn154, 2.47 Å for Ser153, and 2.81 Å and 2.25 Å for Glu33, respectively. Also, three hydrophobic interactions have been observed between ligand L12 and other amino acids within MAPKERK, with bond lengths of 3.24 Å for Val39, 4.84 Å for Leu156, and 4.21 Å for Cys166. Additionally, ligand L12 demonstrates nine van der Waals interactions involving residues Ile84, Gln105, Ala52, Lys54, Asp167, Ile31, Gly34, Gly32, and Ser153. Figure 4 shows detailed 3D and 2D binding interactions of ligand L12 within the active pocket of MAPKERK.

**FIGURE 5:**
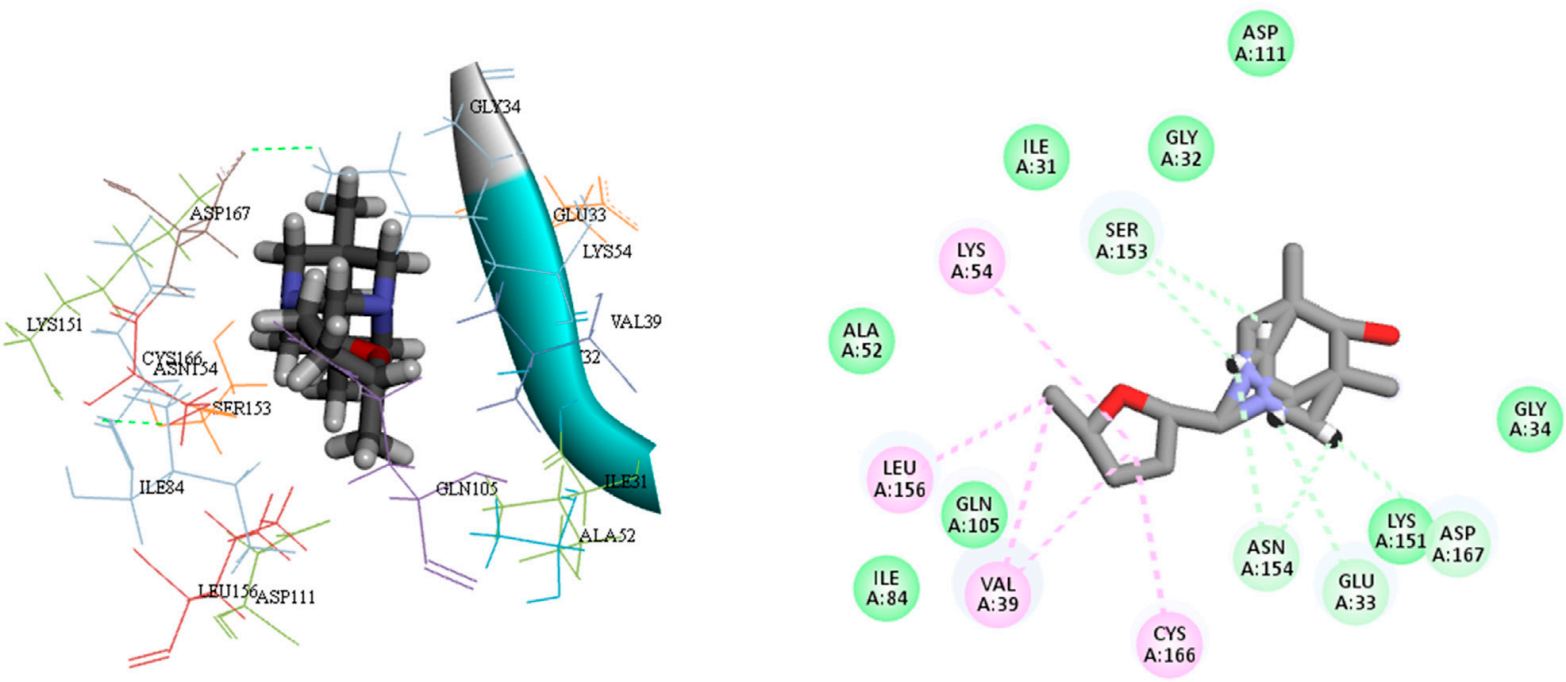
The 3D and 2D bindings mode of **L**_**15**_ into the active site of MAPK^ERK^.

**L**_**15**_ ranked second out of the three top-scoring ligands due to its strong binding interactions with the active site of the MAPK^ERK^ protein. Ligand **L**_**15**_ has six carbon-hydrogen bonds, corresponding to the amino acid residues Ser153 and Glu33, with bond lengths of 2.42 and 2.74, respectively. Additionally, it interacts with Asp167 at a bond length of 3.02 and with Asn154 at bond lengths of 2.18 and 2.23. Furthermore, five hydrophobic interactions have been observed between the ligand L15 and the MAPK^ERK^ protein. These include bond lengths of 4.88 Å for Lys54, 3.82 Å for Cys166, 4.56 Å for Leu156, and two band lengths of 4.60 and 4.85 Å for Val39. Also, ligand **L**_**15**_ interacts with Gly134, Lys151, Gln105, Ile84, Ala52, Ile31, Gly32, and Asp111 in five van der Waals interactions. Figure 5 displays the 2D and 3D binding interactions of **L**_**15**_ within MAPK^ERK^’s active pocket.

**FIGURE 6:**
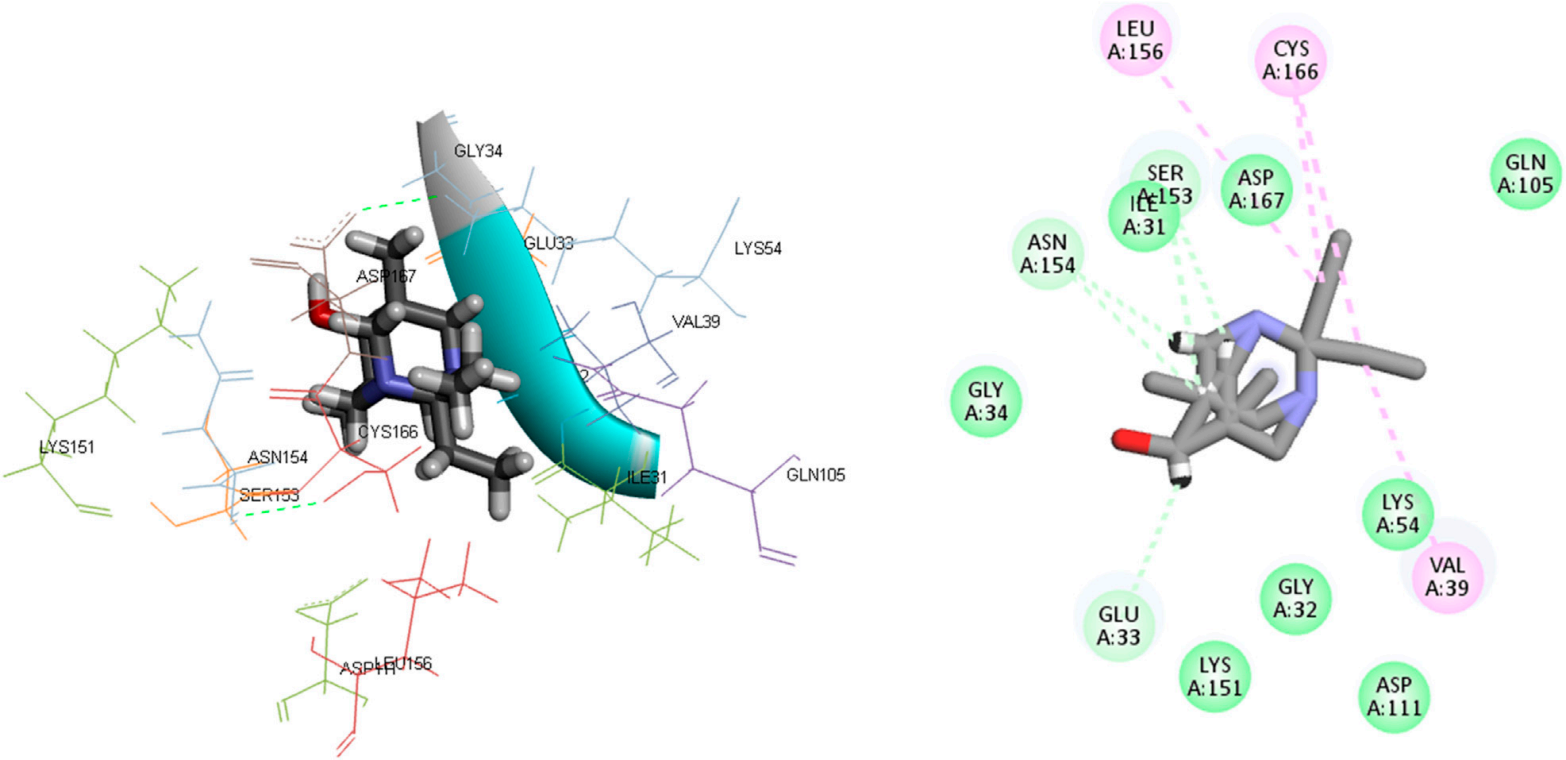
The 3D and 2D bindings mode of **L**_**17**_ into the active site of MAPK^ERK^.

Ligand **L**_**17**_ has five carbon-hydrogen bonds, two of which are associated with amino acid Ser153 and have bond lengths of 2.21 and 2.79, respectively; the other two are related to amino acid Asn154 and have bond lengths of 2.31 and 2.72, respectively; and another is related to Glu33 and has a bond length of 2.48. In addition, it has four alkyl interactions with the residues Leu156, Cys166, and Val39, and nine van der Waals interactions with Gly34, Asp167, Gln105, Asp111, Lys54, Gly32, Lys151, and Ile31. Figure 6 displays the 2D and 3D binding interactions of **L**_**17**_ within MAPK^ERK^’s active pocket. In summary, Table 3 illustrates that the two main interactions in all complexes are hydrophobic intermolecular interactions and carbon-hydrogen bonds.

### 3.3 MD simulations

**FIGURE 7:**
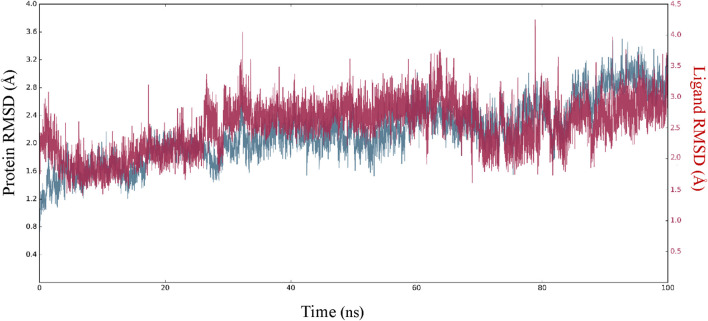
RMSDs of carbon atoms of the protein and **L**_**15**_ through MD simulation.

In order to enhance the reliability of the obtained results and confirm the stability of the system, we combined the docking methods with MD simulation. This allowed us to investigate the changes in the ligand-receptor complex’s conformation during the simulation time (Caporuscio et al., 2011). Simulations offer a detailed investigation of the exact movement of each atom over time, allowing us to investigate changes and fluctuations in protein patterns. MD simulation was conducted on the best-scoring **L**_**12**_ complex for 100 ns. The dynamic stability of the complexes was assessed by measuring the RMSD of the complex backbone atoms during the whole trajectory to confirm their stability (Figure 7). The RMSD curves of the ligand (Lig fit Prot) and the protein backbone (Cα) showed that our simulation had converged and equilibrated after an initial period of disturbance. Throughout the 100 ns simulation, variations in the RMSD of **L**_**12**_ in the binding pocket ranged from 1.70 to 4.20 Å. The MAPK^ERK^ altered its maximum RMSD from 0.85 Å in the starting frame to 3.20 Å. Higher fluctuations were observed between 22 and 30 ns, after which the RMSD remained constant until the last 100 ns. The convergence of RMSD values indicated that **L**_**15**_ and MAPK^ERK^ maintained their interaction through the simulation.

**FIGURE 8:**
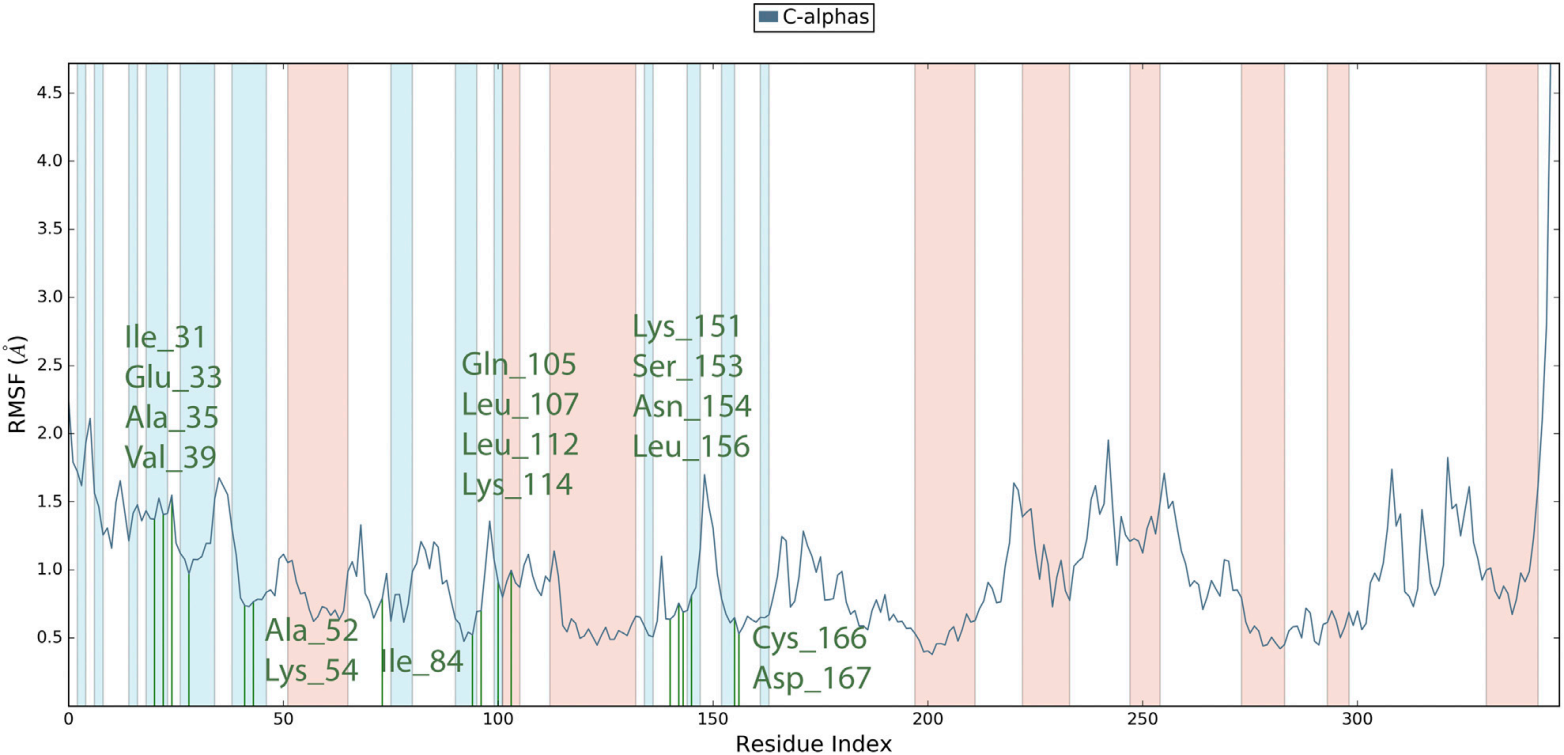
RMSF plot for Cα of MAPK^ERK^ residues in **L**_**12**_-MAPK^ERK^ complex.

Root Mean Square Fluctuation (RMSF) serves as a metric quantifying the average deviation of each atom’s position from its mean position within a specified simulation or ensemble of structures. The RMSF values for the catalytic region of the MAPK^ERK^ of all the complexes exhibited stability. There were no considerable fluctuations observed where the ligand binds to the protein.The comprehensive analysis of the interaction between **L**_**12**_ and the binding site residues of MAPK^ERK^ is illustrated in Figure 8. The residues that interact with **L**_**12**_ are as follows: Ile31,Glu33,Ala35,Val39,Ala52,Lys54,Ile84,Gln105,Leu107,Leu112,Lys114,Lys151,Ser153,Asn154,Leu156,Cys166,Asp167. The residue interacting with **L**_**15**_ is shown in green, while the protein’s secondary structures, helices and β-strands, are represented by orange and blue bands, respectively. The RMSF values for the residues in the binding site were found to be less than 2 Å.

**FIGURE 9:**
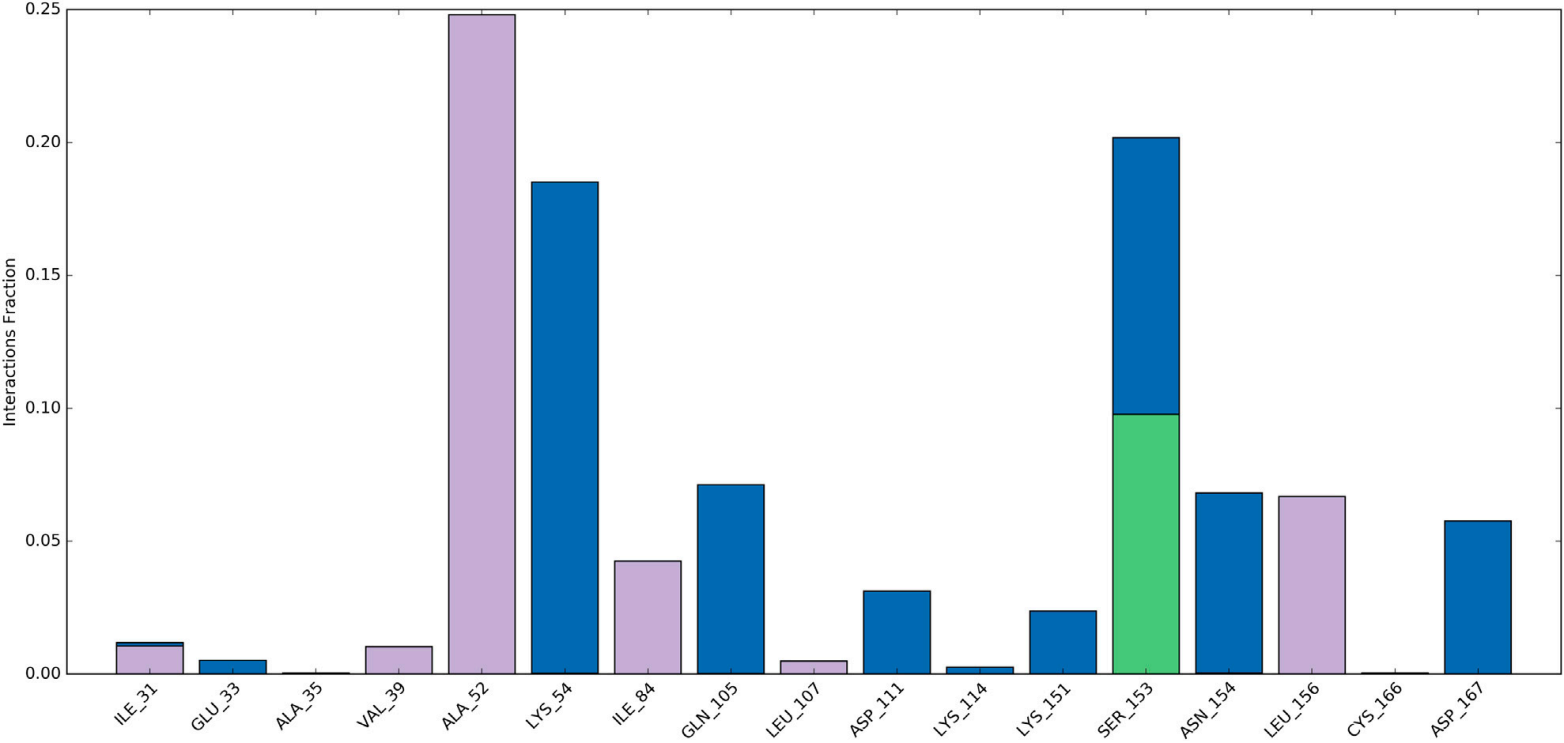
Protein–ligand contacts during simulation.

Ligand-protein interactions can be monitored throughout the simulation. These interactions can be categorized as hydrogen bonds, hydrophobic, ionic, and water bridges, and summarized. As depicted in Figure 9, Ala52, Ile84, and Leu156 engaged in hydrophobic interactions with the ligand for approximately 24%, 4%, and 7% of the simulation duration. Moreover, over at least 3% of the simulation period, Lys54, Gln105, and Asp111, Lys151, Asn154, and Asp167 formed water bridge interactions with the ligand. Significantly, Ser153 demonstrated a variety of interactions, encompassing water-bridged and hydrogen-bond interactions with the ligand. Consequently, this residue experienced numerous interactions throughout the simulation.

**FIGURE 10:**
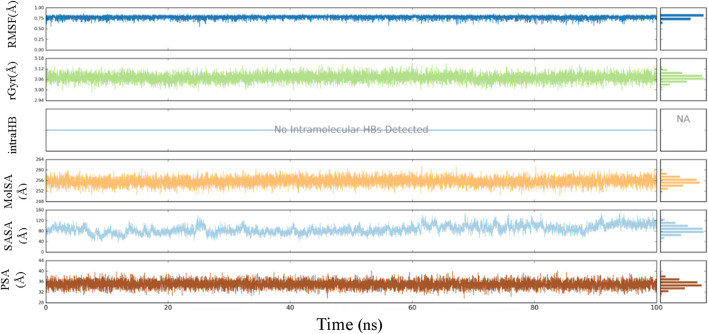
Ligand properties demonstrated by RMSD, rGyr, intraHB, MolSA SASA, PSA.

Throughout the 100-ns MD simulation, ligand properties were examined. The RMSD of a ligand with respect to its reference conformation (time t = 0) was calculated to be between 0.56 and 0.85 Å. The radius of gyration (Rg) provided a means to evaluate the variation in compactness of the ligand-protein complex within the range of 2.97–3.14 Å. The molecular surface area (MolSA) was computed using the van der Waals surface area between 250 and 260 Å. No intramolecular hydrogen bond (intra-HB) was detected. The solvent accessible surface area (SASA) analysis examines the interaction between a ligand and solvents during a 100 ns MD simulation, yielding values ranging from 40 to 158 Å. The analysis also included the examination of the polar surface area (PSA), which provides information about the solvent’s ability to access the surface area given by oxygen and nitrogen atoms. The PSA value ranged from 29 to 40 Å (Figure 10).

### 3.4 Drug likeness and ADMET properties

The physicochemical, pharmacokinetic, and medicinal chemistry features mentioned for our top 18 candidates are shown in Table 4. The above 18 lead ligands were found to have desirable drug-like properties based on Lipinski’s rule of five. The pharmacokinetic parameters revealed that all compounds are highly absorbed after oral administration through the gastrointestinal tract (GI). Structural alarms and pan-assay interference (PAINS) have been utilized in medicinal chemistry to forecast the presence of unstable, reactive, and toxic fragments in a compound’s structure (Baell and Holloway, 2010). All ligands in PAINS descriptors have zero alarms. The synthetic accessibility score (SA score) is a metric used to evaluate the ease of synthesizing drug-like molecules. It was observed that all the compounds possess a favorable SA score, indicating that they can be readily synthesized. The use of CaCo-2 cells, which are generated from human colon epithelial cells, is a widely accepted approach for studying the intestinal absorption of medicines in people. The CaCo-2 cell permeability findings showed that all ligands except **L**_**2**_, **L**_**9**_, **L**_**10**_, **L**_**11**_, **L**_**12**_, **L**_**13**_, **L**_**14**_, **L**_**15**_, **L**_**17**_, and **L**_**18**_ were in the satisfactory range, indicating that these ligands have favorable membrane permeability. In terms of plasma glycoprotein (PGP) substrate, it was observed that, except for ligands **L**_**3**_, **L**_**4**_, **L**_**5**_, and **L**_**11**_, other ligands have inhibitory effects on PGP. As well, all ligands exhibit PGP substrate activity. The computed values for HIA indicate that all ligands except **L**_**12**_, **L**_**14**_, and **L**_**18**_ possess a high likelihood of being effectively absorbed through the intestinal membrane. The assessment of PPB has significance in the evaluation of drug safety. Drugs exhibiting a high PPB value (>90%) are associated with a narrow therapeutic index, whereas those with a low PPB value are considered to be comparatively safer. In the current investigation, it was shown that all ligands exhibited low PPB values. This finding suggests that these particular compounds have a wide therapeutic index, indicating a favorable safety profile. A substance with a positive blood-brain barrier value has a higher lipophilicity profile and may be easily absorbed from plasma membranes. It was noticed that ligand **L**_**1**_ had a higher lipophilicity profile, with values as high as BBB+++. Ligands **L**_**11**_, **L**_**12**_, **L**_**13**_, **L**_**14**_, and **L**_**16**_ are shown to be carcinogenic, while the other ligands are non-carcinogenic. The AMES toxicity profile showed that all ligands have the potential to be toxic. Overall, all ligands demonstrated better ADMET profiles; all values are shown in Table 5.

**TABLE 4:** Physicochemical, pharmacokinetics, and medicinal chemistry properties of the compounds (**L**_**1**_ **-L**_**18**_).

	MW (g/mol)	HBA	HBD	TPSA (Å^2^)	Consensus log Po/w*	MR	GI absorption	BBB Permeant	P-gp substrate	Lipinski	Bioavailability score	PAINS (alert)	Synthetic accessibility score
**L**_**1**_	284.44	2	0	6.48	3.65	96.01	High	Yes	No	Yes	0.55	0	4.44
**L**_**2**_	232.32	3	0	19.62	2.02	73.70	High	Yes	No	Yes	0.55	0	4.66
**L**_**3**_	293.41	3	0	19.37	2.98	96.73	High	Yes	No	Yes	0.55	0	4.52
**L**_**4**_	286.37	4	0	24.94	2.54	87.49	High	Yes	No	Yes	0.55	0	4.81
**L**_**5**_	300.44	3	0	15.71	3.29	97.54	High	Yes	No	Yes	0.55	0	4.51
**L**_**6**_	302.41	4	0	24.94	2.65	94.41	High	Yes	No	Yes	0.55	0	4.59
**L**_**7**_	288.38	4	1	35.94	2.33	89.95	High	Yes	No	Yes	0.55	0	4.45
**L**_**8**_	246.35	3	0	19.62	2.36	78.66	High	Yes	No	Yes	0.55	0	4.88
**L**_**9**_	243.35	3	0	19.37	1.94	79.23	High	Yes	No	Yes	0.55	0	4.30
**L**_**10**_	243.35	3	0	19.37	1.97	79.23	High	Yes	No	Yes	0.55	0	4.25
**L**_**11**_	300.44	3	1	26.71	2.85	97.17	High	Yes	No	Yes	0.55	0	4.67
**L**_**12**_	258.36	3	1	26.71	1.88	82.59	High	Yes	No	Yes	0.55	0	4.33
**L**_**13**_	286.41	3	1	26.71	2.53	92.37	High	Yes	No	Yes	0.55	0	4.55
**L**_**14**_	288.38	4	1	35.94	1.91	89.08	High	Yes	No	Yes	0.55	0	4.50
**L**_**15**_	262.35	4	1	39.85	1.54	79.82	High	Yes	No	Yes	0.55	0	5.02
**L**_**16**_	264.39	3	1	54.95	1.89	80.47	High	Yes	No	Yes	0.55	0	4.76
**L**_**17**_	238.37	3	1	26.71	1.91	77.37	High	Yes	No	Yes	0.55	0	4.68
**L**_**18**_	273.37	4	1	39.6	1.49	85.19	High	Yes	No	Yes	0.55	0	4.62

*Average of five prediction.

**TABLE 5:** ADMET profile of the compounds (**L**_**1**_ **-L**_**18**_).

Absorption and distribution							
Mode	Caco-2 permeability	PGP-Inhibitor	*p*-Glycoprotein substrate (PGPsubstrate)	Human intestinal absorption (HIA)	Plasma protein binding (PPB) (%)	Volume of distribution (VD)	Blood brain barrier (BBB)
**L**_**1**_	−4.95	+++0.980	---0.012	---0.009	84.89	5.016	+++0.997
**L**_**2**_	−5.21	---0.036	---0.013	---0.059	53.52	4.939	+++0.996
**L**_**3**_	−5.01	+++0.990	---0.004	---0.006	83.06	5.128	+++0.996
**L**_**4**_	−4.97	−0.372	---0.006	---0.003	72.43	2.956	+++0.996
**L**_**5**_	−4.76	++0.895	---0.004	---0.003	79.16	4.122	+++0.996
**L**_**6**_	−4.93	---0.059	---0.050	---0.018	74.98	1.904	+++0.995
**L**_**7**_	−5.12	---0.034	---0.006	---0.073	66.49	2.404	+++0.993
**L**_**8**_	−5.02	---0.057	---0.010	---0.012	51.48	4.189	+++0.993
**L**_**9**_	−5.30	---0.049	---0.003	---0.016	22.74	3.306	+++0.991
**L**_**10**_	−5.40	---0.074	---0.005	---0.018	23.57	3.955	+++0.989
**L**_**11**_	−5.23	−0.396	--0.208	---0.022	74.33	4.080	+++0.988
**L**_**12**_	−5.25	---0.037	---0.079	+0.567	31.74	3.436	+++0.986
**L**_**13**_	−5.21	--0.196	--0.131	---0.069	57.29	4.598	+++0.984
**L**_**14**_	−5.26	---0.011	---0.044	−0.390	42.32	3.095	+++0.982
**L**_**15**_	−5.41	---0.004	--0.117	--0.289	44.61	2.423	+++0.966
**L**_**16**_	−4.93	---0.006	---0.009	---0.078	45.22	2.099	+++0.917
**L**_**17**_	−5.39	---0.007	--0.112	---0.018	17.99	1.462	+++0.913
**L**_**18**_	−5.34	---0.009	---0.024	+++0.905	12.22	4.836	++0.827

Metabolism											Elimination	
	P450 CYP1A2 inhibitor	P450 CYP1A2 substrate	P450 CYP3A4 inhibitor	P450 CYP3A4 substrate	P450 CYP2C9 inhibitor	P450 CYP2C9 substrate	P450 CYP2C19 inhibitor	P450 CYP2C19 substrate	P450 CYP2D6 inhibitor	P450 CYP2D6 substrate	T_1/2_	Cl
**L**_**1**_	--0.134	---0.091	---0.004	+0.527	--0.103	−0.306	--0.209	+++0.945	++0.828	++0.893	0.073	7.252
**L**_**2**_	---0.065	---0.075	---0.001	--0.272	---0.057	−0.360	--0.170	+++0.911	+0.527	++0.888	0.558	9.875
**L**_**3**_	−0.366	--0.124	---0.004	−0.378	--0.123	--0.212	−0.323	++0.882	++0.711	++0.888	0.210	5.257
**L**_**4**_	+0.553	--0.109	---0.077	−0.438	---0.075	--0.274	--0.249	+++0.912	++0.899	++0.884	0.238	14.978
**L**_**5**_	--0.148	---0.087	---0.003	−0.421	--0.126	+0.533	−0.315	+++0.940	++0.899	+++0.910	0.112	10.169
**L**_**6**_	---0.042	---0.094	---0.003	+0.595	---0.026	−0.489	--0.108	+++0.936	−0.444	+++0.901	0.400	9.504
**L**_**7**_	---0.051	---0.090	---0.002	--0.289	---0.054	−0.449	--0.123	++0.890	−0.389	++0.884	0.535	12.672
**L**_**8**_	---0.076	---0.087	---0.001	−0.412	---0.040	+0.501	--0.190	+++0.941	+0.568	+++0.901	0.464	7.344
**L**_**9**_	---0.061	--0.101	---0.020	−0.340	---0.100	−0.325	--0.278	++0.893	−0.390	++0.879	0.394	10.831
**L**_**10**_	--0.252	--0.120	---0.052	--0.300	−0.404	+0.571	−0.422	++0.834	+0.620	++0.890	0.370	9.518
**L**_**11**_	---0.069	---0.058	---0.003	−0.418	---0.050	--0.145	--0.118	+++0.939	+0.658	++0.881	0.079	6.663
**L**_**12**_	---0.067	---0.048	---0.001	--0.259	---0.026	---0.095	--0.123	++0.889	+0.560	++0.849	0.200	8.309
**L**_**13**_	---0.080	---0.058	---0.002	−0.307	---0.044	--0.122	--0.148	+++0.927	++0.708	++0.879	0.149	8.565
**L**_**14**_	---0.055	---0.064	---0.002	--0.229	---0.031	−0.382	--0.120	+++0.933	+0.525	++0.899	0.191	8.366
**L**_**15**_	---0.045	---0.056	---0.001	--0.257	---0.013	−0.393	--0.101	+++0.939	−0.396	++0.889	0.392	7.313
**L**_**16**_	---0.063	---0.051	---0.001	--0.207	---0.030	---0.089	--0.105	++0.891	−0.455	++0.886	0.207	5.241
**L**_**17**_	---0.007	---0.041	---0.001	++0.733	---0.001	--0.109	---0.029	+++0.971	−0.458	++0.858	0.154	9.303
**L**_**18**_	---0.042	---0.052	---0.025	--0.263	---0.077	−0.439	--0.105	++0.715	−0.317	++0.851	0.560	11.178

Mode	Toxicity							
	AMES toxicity	Carcinogenicity	Eye corrosion	Eye irritation	hERG	H-HT	LD_50_	Respiratory toxicity
**L**_**1**_	---0.009	++0.765	---0.004	---0.029	---0.054	+0.656	1047.132	+++0.971
**L**_**2**_	---0.009	+++0.940	---0.006	---0.055	---0.011	++0.845	867.161	+++0.978
**L**_**3**_	---0.072	++0.893	---0.003	---0.024	---0.091	++0.882	1112.963	+++0.978
**L**_**4**_	---0.027	+++0.968	---0.003	---0.020	---0.037	++0.851	691.728	+++0.968
**L**_**5**_	---0.009	+++0.929	---0.003	---0.020	---0.074	++0.758	1075.886	+++0.967
**L**_**6**_	---0.012	++0.725	---0.004	---0.020	---0.033	+0.619	749.217	+++0.979
**L**_**7**_	---0.032	++0.821	---0.004	---0.026	---0.021	++0.714	630.931	+++0.973
**L**_**8**_	---0.009	+++0.954	---0.004	---0.043	---0.012	++0.776	992.111	+++0.973
**L**_**9**_	---0.011	+++0.938	---0.004	---0.070	---0.020	++0.846	818.914	+++0.986
**L**_**10**_	---0.017	+++0.915	---0.008	--0.106	---0.021	++0.848	828.397	+++0.989
**L**_**11**_	---0.011	--0.239	---0.003	---0.019	---0.025	--0.127	1020.391	+++0.917
**L**_**12**_	---0.014	−0.336	---0.003	---0.038	---0.012	−0.395	871.432	+++0.916
**L**_**13**_	---0.011	−0.301	---0.003	---0.019	---0.030	--0.236	957.198	+++0.912
**L**_**14**_	---0.010	−0.441	---0.003	---0.022	---0.023	--0.247	707.916	+++0.947
**L**_**15**_	---0.007	++0.875	---0.003	---0.027	---0.007	--0.166	734.321	++0.840
**L**_**16**_	---0.029	+0.688	---0.003	---0.035	---0.009	+0.609	828.422	+++0.955
**L**_**17**_	---0.062	+++0.946	---0.003	---0.075	---0.006	++0.776	1189.216	+++0.988
**L**_**18**_	---0.044	+++0.986	---0.003	---0.027	---0.013	++0.712	680.404	+++0.977

## 4 Conclusion

The study aims to find prospective compounds for MS by targeting the MAPK^ERK^ protein using a computational method to expedite the drug development process and reduce costs. Studies show that heightened MAPK^ERK^ activity in microglial cells associated with MS can lead to inflammation in the CNS and harm oligodendrocytes. In the current study, the findings of molecular docking, ADMET, MD simulation, and DFT calculations suggested that diazaadamantan derivatives can be a suitable scaffold for developing efficient leads capable of inhibiting MAPK^ERK^ and aiding in the battle against MS. During docking with MAPK^ERK^, the **L**_**12**_ receptor exhibited the best binding affinity. A 100 ns MD simulation of the MAPK^ERK^ protein and **L**_**12**_ complex assessed binding stability. The MD simulation showed potent receptor-ligand stability. These ligands’ pharmacokinetic and drug-like properties suggest they can be potential MS therapy options. However, to confirm the biological activity of the selected ligands and assess the value of pharmacological inhibition of MAPK^ERK^, more *in vitro* and *in vivo* research is necessary.

## Data Availability

The original contributions presented in the study are included in the article/Supplementary material, further inquiries can be directed to the corresponding authors.
